# A Proposal of a Method for Ready-Mixed Concrete Quality Assessment Based on Statistical-Fuzzy Approach

**DOI:** 10.3390/ma13245674

**Published:** 2020-12-12

**Authors:** Izabela Skrzypczak, Wanda Kokoszka, Joanna Zięba, Agnieszka Leśniak, Dariusz Bajno, Lukasz Bednarz

**Affiliations:** 1 Faculty of Civil and Environmental Engineering and Architecture, Rzeszow University of Technology, Powstanców Warszawy 12, 35-082 Rzeszow, Poland; izas@prz.edu.pl (I.S.); wandak@prz.edu.pl (W.K.); j.zieba@prz.edu.pl (J.Z.); 2 Faculty of Civil Engineering, Cracow University of Technology, Warszawska 24, 31-155 Kraków, Poland; 3 Faculty of Civil and Environmental Engineering and Architecture, UTP University of Science and Technology, Al. Prof. S. Kaliskiego 7, 85-796 Bydgoszcz, Poland; dariusz.bajno@utp.edu.pl; 4 Faculty of Civil Engineering, Wroclaw University of Science and Technology, Wybrzeże Wyspiańskiego 27, 50-370 Wroclaw, Poland; lukasz.bednarz@pwr.edu.pl

**Keywords:** ready-mixed concrete, construction material, quality assessment, conformity criteria, statistical-fuzzy method

## Abstract

Control of technical parameters obtained by ready-mixed concrete may be carried out at different stages of the development of concrete properties and by different participants involved in the construction investment process. According to the European Standard EN 206 “Concrete–Specification, performance, production and conformity”, mandatory control of concrete conformity is conducted by the producer during production. As shown by the subject literature, statistical criteria set out in the standard, including the method for concrete quality assessment based on the concept of concrete family, continue to evoke discussions and raise doubts. This justifies seeking alternative methods for concrete quality assessment. This paper presents a novel approach to quality control and classification of concrete based on combining statistical and fuzzy theories as a means of representation of two types of uncertainty: random uncertainty and information uncertainty. In concrete production, a typical situation when fuzzy uncertainty can be taken into consideration is the conformity control of concrete compressive strength, which is conducted to confirm the declared concrete class. The proposed procedure for quality assessment of a concrete batch is based on defining the membership function for the considered concrete classes and establishing the degree of belonging to the considered concrete class. It was found that concrete classification set out by the standard includes too many concrete classes of overlapping probability density distributions, and the proposed solution was to limit the scope of compressive strength to every second class so as to ensure the efficacy of conformity assessment conducted for concrete classes and concrete families. The proposed procedures can lead to two types of decisions: non-fuzzy (crisp) or fuzzy, which point out to possible solutions and their corresponding preferences. The suggested procedure for quality assessment allows to classify a concrete batch in a fuzzy way with the degree of certainty less than or equal to 1. The results obtained confirm the possibility of employing the proposed method for quality assessment in the production process of ready-mixed concrete.

## 1. Introduction

The construction industry is an economic sector characterised by high changeability and diversity. Individual character of the facilities constructed is expressed in their unique qualities, such as form, shape and purpose, and influenced by such factors as environmental conditions (the facility’s surroundings), completion time, technologies applied and building materials used. Much of the work related to facility construction involves optimisation of project completion time [1], optimisation of costs [2], energy efficiency [3], which also includes finding optimal technologies [4] and appropriate building materials for the particular project [5]. Execution of construction works within the scheduled time, within the framework of estimated costs and at the assumed quality level is the determinant of success for the investor, the designer and the contractor. The existence of relationship between costs, completion time and project quality, as depicted in the form of project management triangle, is considered to be self-evident [6]. The subject literature provides numerous definitions and interpretations of the term “quality”. Considering the concept of quality in the construction industry, it can be defined as meeting the requirements of the designer, the contractor, the owner and the regulatory agencies [7]. The quality of the facilities constructed is directly influenced by the applied quality control procedures for the execution of construction works at construction sites, and procedures related to the production of building materials in permanent production facilities. As shown in [8], these procedures greatly vary, since measures related to quality control of the execution of construction works can be approached in a relatively flexible manner while remaining within the aforementioned provisions, whereas quality control of the construction materials supplied to the market is strictly regulated.

Concrete is a building material widely used in construction [9], while ready-mixed concrete (RMC) is the principal construction material for civil engineering infrastructure [10]. Currently, the world produces 4.4 billion tons of concrete annually, but that number is expected to rise to over 5.5 billion tons by 2050, according to the Chatham House report [11]. Construction concrete produced under quality control guidelines constitutes about 70% of total concrete production [12]. Since the properties of concrete are shaped from the moment of mixing in a process influenced by many factors, assessment of its quality (parameters) can be carried out at different times: during production, during delivery and before/after construction, and importantly, quality assessment can be performed by different participants of the investment process: the producer, the contractor, and the investor. Achieving the desired quality of concrete involves not only conformity assurance, but also appropriate design of concrete mix and selection of suitable ingredients [13,14,15,16,17,18,19,20,21], proper manufacturing [22,23,24], development of innovative research methods that aid concrete design aimed at obtaining appropriate properties and durability [25,26,27] and development of methods for analysing obtained assessment results both during production and in existing constructions [28,29].

The traditional approach to the quality assessment of ready-mixed concrete is through experiments [30,31], which, however, proves to be both time and resource consuming. The proposed statistical-fuzzy-approach-based method for quality assessment can overcome these limitations. The suggested method may be employed in adaptative neuro-fuzzy inference systems and applied to predict the 28-day compressive strength of concrete for concrete mix design by reducing i.a., the number and scope of trials. The application of the proposed procedure combined with the use of artificial neural networks (ANN) ensures the reliable assessment of concrete compressive strength.

According to the European Standard EN-206 “Concrete–Specification, performance, production and conformity” [32], ready-mixed concrete delivered on the construction site as concrete mix is subject to mandatory control for compliance with the criteria set out by EN-206. The assessment is performed by the producer during production. Other procedures for concrete quality control are mostly optional. It should be underscored that conformity control carried out according to the recommended criteria cannot be regarded as statistical control until objective conclusions are drawn in line with the principles of mathematical statistics. Statistical sample method can raise doubts as to the accuracy of estimation of the concrete property being assessed and the classification of the considered concrete batch. With a small sample size (*n* = 3), as is the case in concrete quality control, qualification errors are not uncommon. Statistical quality control arrangements are a result of a “strategic game” between the producer and the consumer, whereas the standard conformity criteria represent a compromise between the quality, economy and safety requirements. Recommended measures for standard conformity control set out in EN-206 continue to evoke discussions, and the research conducted in the field reveals inadequacies [33,34,35,36,37,38]. These inadequacies concern the analyses of concrete batches conducted before quality assessment and refer to deficiency during continuous production. In view of the above, it would seem justified to seek alternative methods for the quality control of concrete.

In engineering practice, the recognition of the material’s compliance with the specification is decided based on the adopted plan for statistical quality control. It is a standard approach based on binary criteria (met/unmet). This is of particular significance in the case of doubts concerning the quality of material (in this case, ready-mixed concrete) already built into the existing structure, where the material quality is especially tightly linked with the structure’s safety and reliability. For instance, in the case of prestressed structures, both understated and overstated concrete class has a key influence on the fulfilment of the serviceability limit state condition. Accidental understatement of the concrete class may result in the demolition of a structural component or, in extreme cases, an entire structure (e.g., a bridge).

The present paper aims to propose a novel approach to concrete classification based on combining statistical and fuzzy theories as a means of representation of two types of uncertainty: random uncertainty and information uncertainty. In the field of application of statistical decision procedures–in statistical quality control—there are cases of imprecise definition of quality requirements and imprecise assessment of products subject to quality control. Such state of affairs can be caused by various factors of linguistic, economical and statistical nature. Transition from traditional (“hard”) models, with fixed data, relations and limits, to “soft” models that allow some degree of imprecision is made possible by the fuzzy set theory introduced by Zadeh [39].

For the discussed issue of quality control of ready-mixed concrete, a typical situation when fuzzy uncertainty can be taken into consideration is the conformity control of concrete compressive strength, carried out to confirm the declared concrete class. Concrete class is equated with concrete compressive strength (*f_ck_*) and constitutes the basis for evaluating the quality of the concrete produced. The proposed procedure for quality assessment of the concrete produced allows for making effective decisions of two types: non-fuzzy (crisp) or fuzzy, which point out to possible solutions and their corresponding preferences.

### Quality Control of Ready-Mixed Concrete According to EN-206

Quality control of ready-mixed concrete is carried out with the appliance of standard statistical control procedures set out in the European Standard EN-206 “Concrete–Specification, performance, production and conformity” [32]. Conformity control involves applying two conformity criteria:

(1) Individual assessment result criterion *f_ci_*—applied irrespective of the production status (initial or continuous)
  (1) $$f_{ci}\;\geq \;\left(f_{ck}-\;4\right)\;N/{\mathrm{mm}}^{2};$$
(2) Mean assessment result criterion *f_cm_*—applied in three methods depending on the production status:
  – Initial production
    (2) $$f_{cm}\;\geq \;\left(f_{ck}+\;4\right)\;N/{\mathrm{mm}}^{2}\;—\;\mathrm{method}\;A;$$
  – Continuous production
    (3) $$f_{cm}\;\geq \;\left(f_{ck}+\;1.48\cdot \sigma \right)\;N/{\mathrm{mm}}^{2}\;—\;\mathrm{method}\;B;$$
  – The concept of control chart—method C.

Specific details regarding particular methods can be found in [32]. Conformity is confirmed when both criteria are satisfied.

Conformity control of concrete compressive strength is carried out on concretes of specific composition or concrete families. The majority of concrete manufacturers assess the conformity of the concrete produced in accordance with the criteria for initial production, as these criteria are easier to apply and do not require taking into consideration the impact of coefficient of variation/standard deviation of compressive strength. With high heterogeneity of the concrete produced, conformity criteria for continuous production are more rigorous than for initial production, and therefore, most manufacturers apply the conformity criteria (Method A) recommended for *n* = 3.

The conformity criterion for mean compressive strength value and for sample of size *n* = 3, as set out in EN 206 [32], was established according to the following Equations (4) to (7):

(4) $$f_{cm}\geq f_{ck}+k_{1}$$

(5) $$f_{cm}\geq f_{ck}+\left(\frac{k_{1}}{\sigma }\right)\cdot \sigma$$

(6) $$f_{cm}\geq f_{ck}+\lambda^{\prime }\cdot \sigma$$

where

(7) $$\frac{k_{1}}{\sigma }=\lambda^{\prime }$$

and

$k_{1}=4$—test coefficient value set out by the standard [28],
σ—standard deviation for population.

As proposed by Taerwe [34] and set out in EN 206 [32], the values of λ′ for correlated results are given as follows (see Table 1):

For initial production, the standard conformity criterion was established for constant standard deviation of 4/2.67 = 1.5 MPa, irrespective of mean compressive strength value.

Applying the conformity criteria set out in EN 206 [28] for a sample of size *n* = 3 (Method A) without providing the standard deviation value may contribute to the deterioration in concrete quality and, in consequence, lead to an excessive recipient risk [35,36,37].

**Figure 1:**
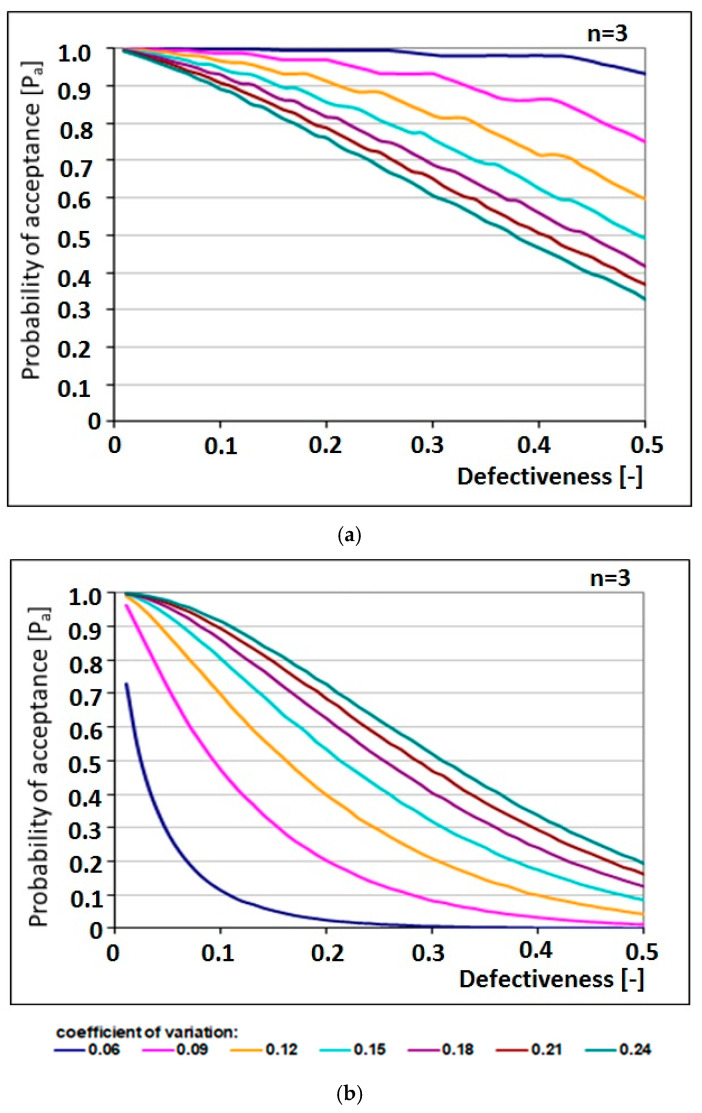
OC curves for conformity criteria for samples of sizes *n* = 3 and normal distribution of concrete compressive strength: for a criterion for (**a**) individual results and (**b**) mean value.

This is confirmed by the results of random simulations and the analysis of conformity criteria for a sample of size *n* = 3, performed by means of operating characteristic (OC) curves [35]. On the basis of these operations, the following conclusions can be formulated (Figure 1):

- The concrete acceptance probability is not always a compromise between the producer risk and the customer risk. Applying the standard conformity criteria may lead to an excessive customer risk, especially in the case of an assumption of log-normal distribution of compressive strength.
- Applying the standard conformity criteria may lead the producer to adopting strategies involving higher production costs, as it can unnecessarily require higher mean values of production with higher standard deviations. These criteria are not recommended for production with small deviation and may be a reason for concealing the results for samples of understated compressive strength.
- Applying the standard conformity criteria may produce too high values of the consumer risk.

**Figure 2:**
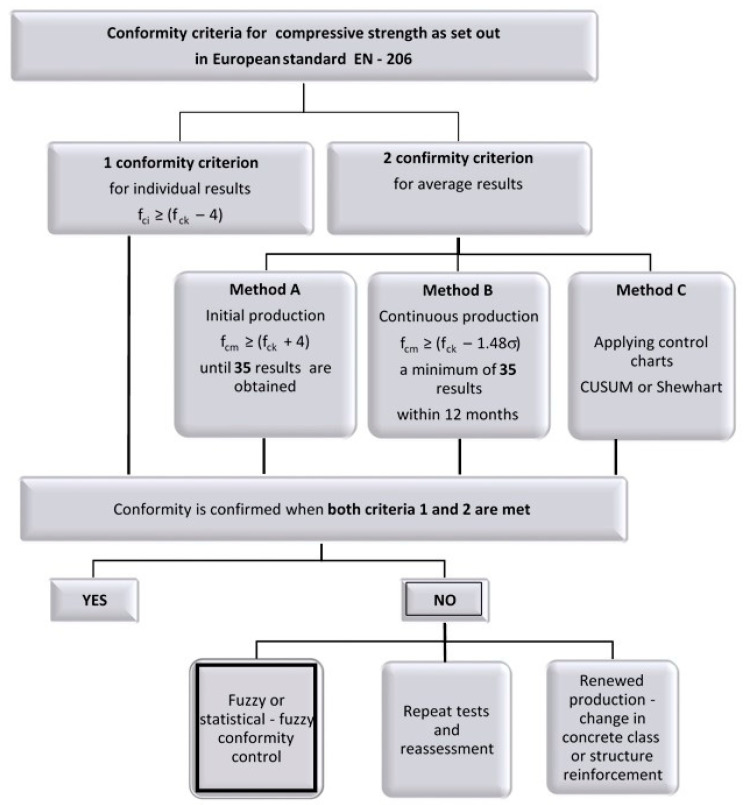
Conformity control of concrete compressive strength according to EN 206 [32], where *f_cm_* is the mean compressive strength of concrete, *f_ck_* is the characteristic compressive strength of concrete, and σ is the estimate for the standard deviation of a population.

Statistical-fuzzy methods of conformity control could be applied as tools supporting initial production. Assessing the concrete class by determining the degree of certainty of concrete belonging to the class intended at the design stage could be an effective tool in decision-making in view of uncertainties related to concrete classification. The place of the proposed method in the conformity control process is presented in Figure 2.

## 2. Materials and Methods

### 2.1. Conformity Control of Concrete Compressive Strength in Consideration of Measurement Uncertainty

Conformity criteria set out in EN 206 [32] and other conformity criteria given in technical specification of products all assume that the assessment results obtained are free of measurement uncertainty—which is not true. Each of these values is burdened with measurement “errors” of type I and II. An assessment result is an approximation of the value measured and should be presented along with measurement uncertainty.

As required by ISO/IEC 17,025 [40], it is necessary for all accredited laboratories to specify measurement uncertainty. Every assessment result is, therefore, not a value but an interval, and should be presented with measurement uncertainty taken into account. When relating the assessment result to the conformity criteria set out in standard [32], it is not particular results but intervals that are subject to analysis. Such an analysis was carried out for the purpose of the present paper.

**Figure 3:**
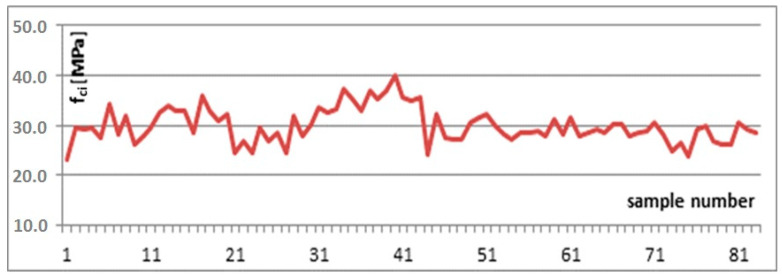
Compressive strength assessment results.

The analysis concerned a population of assessment results for concrete of identical composition, produced by the same concrete batching plant. The concrete analysed was assumed to be of class C20/25 and was characterised by high defectiveness. For the purpose of the analysis, the same criteria were adopted for initial production and overlapping assessment results. The population of results analysed is presented in Figure 3.

In the case analysed, the conformity criterion concerning particular values did not present a hazard for concrete classification in terms of its compliance with the standard (Table 2). All of the results obtained were higher than required to meet this criterion. For conformity control, the criterion related to the mean value was decisive in approving the concrete batch assessed.

The population of results analysed was encumbered with an 8-percent error bias. In the case analysed, 42 percent of assessment results did not meet the standard conformity criteria for initial production. As the compressive strength assessment and sampling were conducted by an accredited laboratory, it was possible to establish the value of measurement uncertainty for defining compressive strength. Measurement uncertainty was estimated at 1.1 MPa. With this assumption, bounds of the result intervals were calculated and compared with the standard conformity criteria related to the mean value. With measurement uncertainty taken into account, the number of results that did not meet the standard conformity criteria decreased to 19 percent. In the example presented, the measurement uncertainty of the results obtained is low in relation to the compressive strength values obtained. Even with such a low level of measurement uncertainty, taking it into account in conformity analysis allows for reducing the number of non-compliant results by over 50 percent.

Having analysed the same results according to the criteria for continuous production, it can be observed that about 52 percent of the results do not meet the standard conformity criteria [19]. With measurement uncertainty taken into consideration, the number of non-compliant results is reduced to about 38 percent. This confirms that in the case of high variability of the quality of concrete (standard deviation of the population of results amounting to 3.5 MPa), it is inadvisable to conduct quality control according to the criteria for continuous production.

### 2.2. Alternative Conformity Criteria for Concrete Compressive Strength

Formulating the statistical conformity criteria for concrete compressive strength remains a complicated issue due to the difficulties related to the insufficiency of statistical methods for small size samples (*n* < 15) and initial production, particularly for samples of size less than or equal to 6.

Employing statistical-fuzzy methods to verify the conformity of a concrete batch might increase the effectiveness of the quality assessment of the concrete produced. Fuzzy functions might be applied on the basis of expertise or marginal distribution parameters (mean and standard deviation) for the considered concrete class and adjacent concrete classes [18,20].

While assessing the quality of the concrete produced, the results of the verification of compliance of concrete compressive strength might be considered as random events, whereas the conformity criteria can be regarded as fuzzy limit values. Conformity criteria for compressive strength, which constitute the basis for the assessment of concrete quality, might be represented as a probability for a random event to be found in a region with fuzzy limits (after Zadeh [39]) or a fuzzy number of known membership function corresponding to the probability that the event belongs to a certain interval [37].

The compressive strength (*f_c_*) of concrete that complies with the conformity criterion can be represented as a fuzzy set (8):

(8) $$T=\left[f_{cm},\;\mu \left(f_{cm}\right)\right]\;|f_{cm}\in T,\;\mu \left(f_{cm}\right):T\to \left[0,1\right]$$

where $\mu_{f_{C}}\left(f_{cm}\right)$ is a membership function that assigns each element of compressive strength set $f_{cm}\in T$ a degree of belonging to fuzzy set *f_c_* in interval [0, 1].

Classification of the considered concrete batch into a specific class generally depends on the fulfilment of the condition related to mean compressive strength in sample, *f_cm_* (Figure 1b). Sporadically, the condition concerning particular test results *f_ci_* is the decisive condition for the fulfilment of the conformity criteria (Figure 1a) [34,35,37,38,41]. Since statistical conformity criteria are found to be insufficient, statistical-fuzzy methods can be applied to define class membership functions, and both standards and expertise can be taken into consideration in the quality control of the concrete produced.

Standard conformity criteria for concrete compressive strength can be given in Equations (9) and (10):

– For method A and sample of size *n* = 15, (9):
  (9) $$\;\;f_{cm}\geq f_{ck}+4\;\to T\;$$
  where *f_cm_* is the mean compressive strength of concrete, and *f_ck_* is the characteristic compressive strength of concrete.
– For method B and sample of size *n* ≥ 15, (10):
  (10) $$f_{cm}\;\geq f_{ck}+1.48\sigma \;\to T$$
  where *f_cm_* is the mean compressive strength of concrete, *f_ck_* is the characteristic compressive strength of concrete, *σ*-estimate for the standard deviation of a population.

In Equations (6) and (7), the test characteristic T is a fuzzy value of membership function μ_T_(t) that can be determined for specific concrete classes on the basis of a statistical-fuzzy experiment.

In order to determine the membership function for the considered concrete classes (three adjacent concrete classes), statistical-fuzzy method (three-phase method) was applied [42,43]. The method proposed elaborates on the concept by Woliński [43].

The statistical-fuzzy conformity control procedure of concrete compressive strength consists of two stages. The first stage is to determine marginal distribution parameters, and for that purpose, random variables *x* and *y* were defined. The variable *x* represents the point of division of the values of test characteristics *T* for the considered concrete class and lower. The variable *y* represents the point of division of test characteristics for the considered concrete class and higher. It is assumed that the pair *(x*, *y)* is a two-dimensional, normal random variable, for which marginal distributions *p_x_(t)* and *p_y_(t)* of random variables *x* *→N(m_x_*,*σ_x_)* and *y* *→ N(m_y_*,*σ_y_)* may be determined. Marginal distribution parameters were determined by means of Monte Carlo simulation methods and the following calculation algorithm [37,44]:

1. Generate *N* groups of random numbers of size *n* = 3 from normal distribution;
2. Randomly select concrete class—Concrete of three adjacent classes *C_i_*_−__1_, *C_i_*, *C_i_*_+1_ (identical probability of 1/3);
3. Randomly select standard deviation from 2, 3, 4, 5, 6 MPa with 1/5 probability;
4. Repeat (1) and (2) *n*-times to obtain *f_ci_*,…, *f_cn_*;
5. Randomly select defectiveness *w* from normal distribution;
6. Calculate mean compressive strength of adjacent concrete classes from Equation (11):
  (11) $$f_{cm(C_{i-1},C_{i})}=\frac{m_{C_{i-1}}+m_{C_{i}}}{2}\;\;\mathrm{and}\;\;f_{cm}{}_{\left(C_{i},C_{i+1}\right)}=\frac{m_{C_{i}}+m_{C_{i+1}}}{2}$$
7. Calculate standard deviation from Equation (12):
  (12) $$s_{\left(C_{i-1},C_{i}\right)}=\frac{1}{n}\sqrt{(s_{C_{i-1}}{}^{2}+s_{C_{{}_{i}}}{}^{2}}\;\;\mathrm{and}\;\;s_{\left(C_{i},C_{i-1}\right)}=\frac{1}{n}\sqrt{(s_{C_{1}}{}^{2}+s_{C_{{}_{i-1}}}{}^{2}}$$
8. Determine the characteristic compressive strength for the considered and lower concrete classes from Equation (13):
  (13) $$f_{ck(C_{i-1},C_{i})}=m_{\left(C_{i-1},C_{i}\right)}-t(w)s_{\left(C_{i-1},C_{i}\right)}$$
  and for the considered and higher concrete classes from Equation (14):
  (14) $$f_{ck(C_{i},C_{i+1})}=m_{\left(C_{i},C_{i+1}\right)}-t(w)s_{\left(C_{i},C_{i+1}\right)}$$
9. Calculate mean compressive strength of the considered and lower concrete classes from Equation (15):
  (15) $$f_{cm}{}_{\left(C_{i-1},C_{i}\right)}=f_{ck(C_{i-1},C_{i})}+4$$
  and of the considered and higher concrete classes from Equation (16):
  (16) $$f_{cm}{}_{\left(C_{i-1},C_{i}\right)}=f_{ck(C_{i},C_{i+1})}+4$$
10. Create a table for the probability distribution function of random vector (*ξ, η)* and determine the histogram of marginal distributions by summing rows and columns. The first marginal distribution is the sum of rows and the classification by the considered and lower concrete classes. The second marginal distribution is the sum of columns and the classification by the considered and higher concrete classes.

The obtained graphs of marginal distribution probability functions *p_ξ_(x_n_)* and *p_η_(x_n_)* (marginal distribution parameters) are the basis for determining membership functions of test characteristics for specific concrete classes, i.e., the second stage of calculations.

The calculations were performed in accordance with the adopted algorithm. The membership function of the test characteristic *T_i_* for the considered *i*-class of concrete and higher can be represented by Equation (17):

(17) $$\mu_{Ci}\left(f_{cm}\right)=\int_{-\infty }^{f_{cm}}p_{\eta }\left(f_{cm}\right)df_{cm}=F\left(\frac{f_{cm}-m_{\eta }}{\sigma_{\eta }}\right)$$

whereas the membership function of the test characteristic *F_i_* for the considered *i*-class of concrete and higher can be expressed by the following Equation (18):

(18) $$\mu_{Ci-1}\left(f_{cm}\right)=\int_{f_{cm}}^{+\infty }p_{\xi }\left(f_{cm}\right)df_{cm}=1-F\left(\frac{f_{cm}-m_{\xi }}{\sigma_{\xi }}\right)$$

The fuzzy membership function for the considered *i*-class of concrete *f_ci_* can be calculated from Equation (19) or (20):

(19) $$\mu_{Ci+1}\left(f_{cm}\right)=1-\int_{f_{cm}}^{+\infty }p_{\xi }\left(f_{cm}\right)df_{cm}-\int_{-\infty }^{f_{cm}}p_{\eta }f\left(f_{cm}\right)$$

(20) $$\begin{array}{l} \mu_{Ci+1}\left(f_{cm}\right)=1-\left[1-F\left(\frac{f_{cm}-m_{\xi }}{\sigma_{\xi }}\right)\right]-F\left(\frac{f_{cm}-m_{\eta }}{\sigma_{\eta }}\right) \end{array}$$

Eventually, Equation (20) can be written the following Equation (21):

(21) $$\mu_{Ci+1}\left(f_{cm}\right)=F\left(\frac{f_{cm}-m_{\xi }}{\sigma_{\xi }}\right)-F\left(\frac{f_{cm}-m_{\eta }}{\sigma_{\eta }}\right)$$

where *F(z)* is a Laplace function given by Equation (22):

(22) $$F\left(z\right)=\frac{1}{\sqrt{2\pi }}\int_{-\infty }^{z}exp(-0.5z^{2})dz$$

Having calculated membership functions for different concrete classes (considered concrete class and adjacent concrete classes) and mean compressive strength for the sample of size n, one may determine the degree of concrete belonging to a specific concrete class. Based on the *μ_K_*(*f_cm_*) value, the considered concrete batch can be recognized as a specific concrete class. Such recognition might be more or less accurate, depending on the economic requirements and the impact of classification on the quality assessment of the concrete produced.

### 2.3. Example of Application of the Statistical-Fuzzy Conformity Criteria for Concrete of Class C20/25

The procedure of statistical-fuzzy conformity control (Section 2.2) was carried out for concrete of class C20/25. By generating 100,000 groups of random numbers of size *n* = 3, consistent with normal distribution, marginal distribution density functions and fuzzy membership functions were estimated for concrete class C25/30 and every second adjacent concrete class, C16/20 and C25/30.

**Figure 4:**
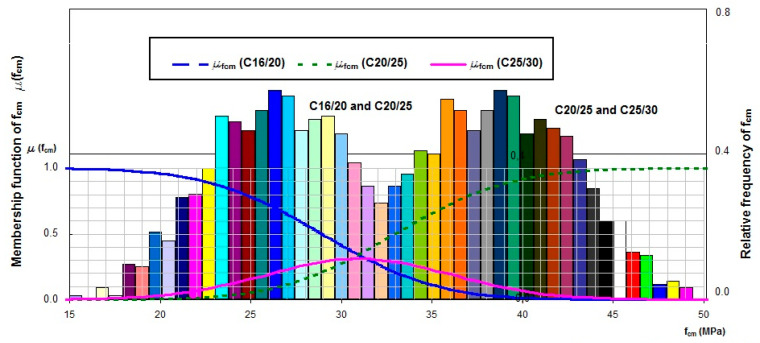
Marginal distribution and membership functions for C20/25 and every adjacent concrete class: C16/20 and C25/30.

The analysis was carried out for concrete of class C20/25 with the following resulting parameters of marginal distribution of random variable x →N(*m_x_,σ_x_*), i.e., the point of division for concrete of classes C16/20 and C20/25, *m_x_* = 26.5 MPa, and *σ_x_* = 4.48 MPa, respectively. The parameters of marginal distribution of random variable y → N(*m_y_,σ_y_*), the point of division for concrete of classes C20/25 and C25/30, were estimated as *m_y_* = 39.8 MPa and *σ_y_* = 5.46 MPa, respectively (Figure 4).

**Figure 5:**
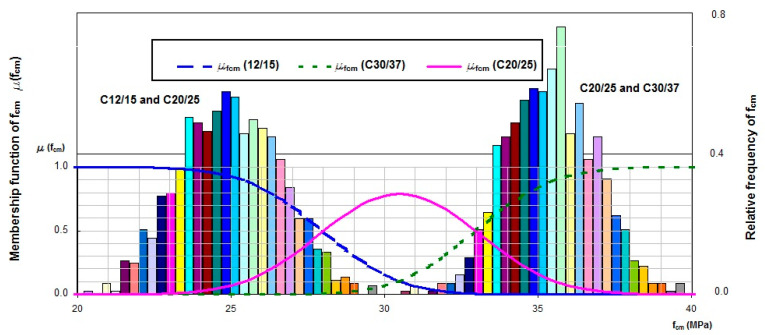
Marginal distribution and membership functions for C20/25 and every second adjacent concrete class: C12/15 and C30/37.

The density functions overlap, indicating that the number of classes proposed by the standard is too high, which makes it difficult to classify a concrete batch to a specific class. Irrespective of mean compressive strength value, the membership function graph (green curve) for the considered concrete class C20/25 does not reach value of 1.0, which allows for concluding that the recommended concrete class division is too dense. The above analysis was carried out for concrete class C20/25 and every second adjacent concrete class (Figure 5).

Marginal distribution graphs for the considered concrete class C20/25 and every second adjacent class, C12/15 and C30/37, also overlap, but the maximum abscissa value of the membership function for the considered concrete class C20/25 amounts to 0.83. By performing subsequent calculations, membership functions for separate concrete classes would be obtained, marginal distributions would not overlap, and the membership function graph (green curve) for the concrete class C20/25, for specified values of mean compressive strength, would reach the value of 1.0.

In accordance with Figure 5, an assessment of a concrete batch was carried out for the statistical-fuzzy conformity criterion developed following the algorithm described above. The concrete batch was assessed based on a sample of size *n* = 3 of concrete class C20/25. Mean compressive strength is 30.5 MPa. On the basis of the membership functions determined (Figure 5), it can be concluded that the concrete batches for which mean compressive strength from the sample test amounts to 30.5 MPa can be classified as class C20/25 with a 0.8 degree of certainty. Concrete batches of mean compressive strength from interval (28.0; 30.8) MPa can be classified as class C20/25 or C12/15 with a degree of certainty from 0.5 to 0.8, respectively. Concrete batches of mean compressive strength from interval (30.8; 33.0) MPa can be classified as class C30/37 with a degree of certainty from 0.8 to 0.5.

## 3. Results and Discussion

The applied statistical-fuzzy methods of concrete classification showed that the concrete classification recommended by the standards includes too many concrete classes of overlapping density distributions (see Figure 4 and Figure 5). Irrespective of mean compressive strength value, the membership function graph plotted for the considered concrete class C16/20 does not reach value of 1.0, which allows for concluding that the recommended concrete class division is too dense. The standards recommended by EN 206 [32] are “too vague” and may lead to understating or overstating concrete class and to concealing the results of understated compressive strength.

Furthermore, when applying the concept of concrete family, standard conformity criteria can conceal the results of understated compressive strength. With the use of the concept of concrete family, small concrete production plants are able to assess the conformity of a larger number of concrete mixes with the benefit for both manufacturer and recipient. Theoretically, the manufacturer can improve the quality of concrete and detect changes in concrete production more quickly, so that the recipient could be informed of the quality of the finished product. What raises doubts is combining the results for different concrete classes of the same family. The results are combined and tested collectively, and as a result, “bad” results (low compressive strength) can be masked by “good” results (high compressive strength). With regard to the concrete family, it is necessary to apply: a single cement type, a single concrete class, aggregate of similar characteristics (granulation, mineralogical composition, geological origin), concretes with or without additions, all consistencies, concretes of limited range of compressive strength.

**Figure 6:**
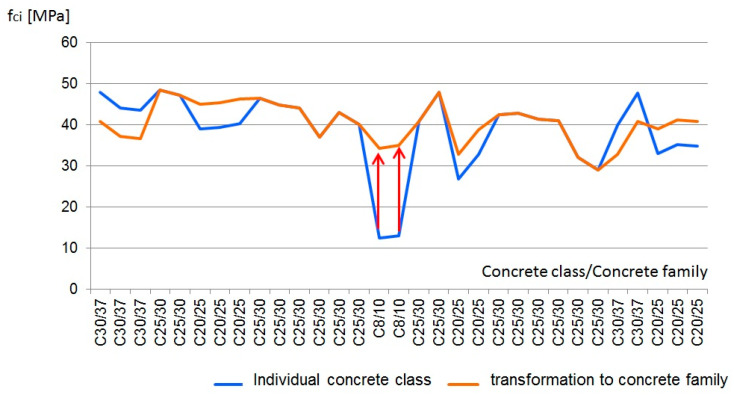
Real results of compressive strength assessment and values for particular classes transformed in relation to the referential concrete in the concrete family.

EN 206 [32] standard does not specify the range of compressive strength. When considering the concrete family composed of four concrete classes: C8/10, C20/25, C25/30 and C30/37, it may be concluded that combining all four classes, i.e., a wide range of classes, is not an appropriate practice. Low values of compressive strength are masked by high values of compressive strength of referential concrete (Figure 6) through transformation and application of the proportionality principle-based method in compliance with CEN CR 13,901 report [45].

Therefore, concretes of a limited range of compressive strength should be applied with regard to the concrete family. In accordance with the statistical-fuzzy analysis carried out, it is recommended to limit the range of compressive strength to three adjacent classes so as to ensure the effectiveness of the conformity control performed for the concrete family.

The statistical-fuzzy methods proposed can be applied in cases of non-compliance with the concrete class intended by the design. The decision of either demolition or reinforcement of a structure may be preceded by the fuzzy concrete classification analysis, whose results may impact both the designer’s and investor’s decisions related to the state of the structure analysed [46].

Taking into account the compressive strength measurement uncertainty broadens the range of acceptability of assessment results obtained. It is in the interest of each party of the construction process for a reliable assessment of concrete conformity to be performed.

In the analysis carried out during conformity assessment, it is important to consider that each result obtained is encumbered with uncertainty, thus disregarding uncertainty completely is not an appropriate approach. The only case when it is possible to disregard measurement uncertainty in assessment is a situation when all of the results obtained meet the conformity criteria. In other instances, i.e., when the product is disqualified on the basis of the results obtained without measurement uncertainty taken into account, such an approach is unadvisable, as it may lead to a falsely negative result for a product that, in fact, meets the standard conformity criteria.

## 4. Conclusions

Taking into account the compressive strength measurement uncertainty broadens the range of acceptability of assessment results obtained. It is in the interest of each party in the construction process that a reliable assessment of concrete conformity be performed.

In the analysis carried out during conformity assessment, it is important to consider that each result obtained is encumbered with uncertainty, and thus, disregarding uncertainty completely is not an appropriate approach. The only case when it is possible to disregard measurement uncertainty in assessment is a situation when all of the results obtained meet the conformity criteria. In other instances, i.e., when the product is disqualified on the basis of the results obtained without measurement uncertainty being taken into account, such an approach is unadvisable, as it may lead to a falsely negative result for a product that, in fact, meets the standard conformity criteria.

Standard conformity criteria and procedures for assessing the compressive strength of concrete and verifying the concrete’s compliance with the requirements set for designed concrete classes frequently lead to inappropriate production-related decisions and strategies. Doubts regarding the assessment and classification of the compressive strength of concrete are, therefore, the reason for seeking new methods based on statistical-fuzzy procedures supporting the quality control of the concrete produced. Statistical-fuzzy methods are, therefore, proposed as an alternative in the quality assessment of ready-mixed concrete:

- The proposed concept of quality assessment allows for minimising the risk of wrong classification of a concrete batch, i.e., overstating or understating the concrete class.
- Employing non-standard methods of conformity control of concrete compressive strength may become a useful tool in the investment-related (technology-related) decision-making process.
- The analyses carried out reveal that the statistical-fuzzy conformity control can play an arbitrary role in the quality assessment of the concrete produced.
- Statistical-fuzzy and fuzzy methods allow to take into account the opposing requirements of safety, quality and economy. Taking these requirements into consideration is made possible by determining a degree of membership lower than 1 for the considered concrete class.
- The alternative method of concrete quality assessment is easy to apply; however, it requires a complex calculation procedure, which significantly limits its universal use in the production process. Widespread application of this method would require implementing specialised utility software developed based on specific algorithms.
- The advantages of the statistical-fuzzy approach are particularly observable when employing the concept of concrete families. It allows to minimise the uncertainty connected to the transformation relation between the results for compressive strength of each concrete family member.
- Based on this approach, a risk matrix may be developed for a construction facility in order to verify the assigned reliability class specified in the construction design.
- Statistical-fuzzy methods are fully compatible with the concept of sustainable construction. Accidental understating of the concrete class results in the rejection of a concrete batch by the recipient. An unsuitable concrete mix is then considered as construction waste, which contradicts the principles of rational use of construction materials and mineral resources.

## Figures and Tables

**Table 1:** λ’ values for correlated results of mixed size samples [34].

Number (*n*) of Results	Value λ′
3	2.67
15	1.48

**Table 2:** Fragment of the table presenting the conformity assessment of the population of results analysed.

Number Sample	Compressive Strength	Criterion 1	Assessment	Criterion 2	Assessment	Compressive Strength + Uncertainty	Criterion 2 + Uncertainty	Assessment
[-]	*f_ci_* [MPa]	*f_ci_* [MPa]	[-]	*f_cm_* [MPa]	[-]	*f_ci_* [MPa]	*f_ci min_* [MPa]	[-]
1	23.1	23.1	met	-		24.2	-	
2	29.7	29.7	met	-		30.8	-	
3	29.1	29.1	met	27.3	unmet	30.2	28.4	unmet
4	29.5	29.5	met	29.4	met	30.6	30.5	met
5	27.5	27.5	met	28.7	unmet	28.6	29.8	met
6	34.3	34.3	met	30.4	met	35.4	31.5	met
7	28.1	28.1	met	30.0	met	29.2	31.1	met
8	31.9	31.9	met	31.4	met	33.0	32.5	met
9	26.1	26.1	met	28.7	unmet	27.2	29.8	met
10	28.0	28.0	met	28.7	unmet	29.1	29.8	met
11	29.4	29.4	met	27.8	unmet	30.5	28.9	unmet
12	32.6	32.6	met	30.0	met	33.7	31.1	met
13	33.8	33.8	met	31.9	met	34.9	33.0	met
14	33.0	33.0	met	33.1	met	34.1	34.2	met
15	32.8	32.8	met	33.2	met	33.9	34.3	met
